# Novel Biomarkers for Limb Girdle Muscular Dystrophy (LGMD)

**DOI:** 10.3390/cells13040329

**Published:** 2024-02-10

**Authors:** Sara Aguti, Gian Nicola Gallus, Silvia Bianchi, Simona Salvatore, Anna Rubegni, Gianna Berti, Patrizia Formichi, Nicola De Stefano, Alessandro Malandrini, Diego Lopergolo

**Affiliations:** 1 Department of Medicine, Surgery and Neurosciences, University of Siena, 53100 Siena, Italy; sara.aguti@ucl.ac.uk (S.A.); gallus2@unisi.it (G.N.G.); silviabianchi.si@gmail.com (S.B.); dr.simona.salvatore@gmail.com (S.S.); gianna.berti@unisi.it (G.B.); patrizia.formichi@unisi.it (P.F.); destefano@unisi.it (N.D.S.); alessandro.malandrini@unisi.it (A.M.); 2 UOC Neurologia e Malattie Neurometaboliche, Azienda Ospedaliero-Universitaria Senese, Policlinico Le Scotte, Viale Bracci, 16, 53100 Siena, Italy; 3 Molecular Medicine for Neurodegenerative and Neuromuscular Disease Unit, IRCCS Stella Maris Foundation, 56128 Pisa, Italy; anna.rubegni@fsm.unipi.it

**Keywords:** *CAPN3*, limb girdle muscular dystrophy, western blotting analysis, RNA

## Abstract

Objective: To identify novel biomarkers as an alternative diagnostic tool for limb girdle muscular dystrophy (LGMD). Background: LGMD encompasses a group of muscular dystrophies characterized by proximal muscles weakness, elevated CK levels and dystrophic findings on muscle biopsy. Heterozygous *CAPN3* mutations are associated with autosomal dominant LGMD-4, while biallelic mutations can cause autosomal recessive LGMD-1. Diagnosis is currently often based on invasive methods requiring muscle biopsy or blood tests. In most cases Western blotting (WB) analysis from muscle biopsy is essential for a diagnosis, as muscle samples are currently the only known tissues to express the full-length *CAPN3* isoform. Methods: We analyzed *CAPN3* in a cohort including 60 LGMD patients. Selected patients underwent a complete neurological examination, electromyography, muscle biopsy, and skin biopsies for primary fibroblasts isolation. The amount of CAPN3 was evaluated by WB analysis in muscle and skin tissues. The total RNA isolated from muscle, fibroblast and urine was processed, and cDNA was used for qualitative analysis. The expression of *CAPN3* was investigated by qRT-PCR. The CAPN3 3D structure has been visualized and analyzed using PyMOL. Results: Among our patients, seven different *CAPN3* mutations were detected, of which two were novel. After sequencing *CAPN3* transcripts from fibroblast and urine, we detected different *CAPN3* isoforms surprisingly including the full-length transcript. We found comparable protein levels from fibroblasts and muscle tissue; in particular, patients harboring a novel *CAPN3* mutation showed a 30% reduction in protein compared to controls from both tissues. Conclusions: Our findings showed for the first time the presence of the *CAPN3* full-length transcript in urine and skin samples. Moreover, we demonstrated surprisingly comparable CAPN3 protein levels between muscle and skin samples, thus allowing us to hypothesize the use of skin biopsy and probably of urine samples as an alternative less invasive method to assess the amount of CAPN3 when molecular diagnosis turns out to be inconclusive.

## 1. Introduction

Limb girdle muscular dystrophy (LGMD) includes a group of muscular dystrophies showing proximal muscle weakness, elevated levels of creatine kinase (CK) and dystrophic changes on muscle biopsy. Actually, more than 30 recessive and dominant inherited types of LGMD have been described [1,2]. Biallelic *CAPN3* mutations can cause autosomal recessive LGMD-1, while heterozygous mutations are associated with autosomal dominant LGMD-4. *CAPN3* encodes for the protein Calpain 3 (CAPN3, calcium-activated neutral proteinase 3). CAPN3 belongs to the Ca^2+^-dependent non-lysosomal calpain cysteine protease family; it is a skeletal muscle-specific isoform that associates with titin [3] and filamin C [4]. The function of calpain 3 is to date not fully elucidated. Nearly 500 CAPN3 mutations have been described thus far, with the majority affecting the protein’s catalytic activity, while others have profound impacts on its interactions with other proteins, including titin [5].

It may be challenging to make a differential diagnosis between LGMD-1 patients and patients with recessive LGMDs; however, clinical hallmarks of LGMD-1 can include the following: (a) absence of heart disease; (b) asymmetric muscle weakness and atrophy [6,7]; and (c) early scapular winging. In fact, the involvement of an upper extremity may be the first sign in some patients. Muscle MRI usually shows dystrophic changes in the posterior thigh muscles and the adductor magnus muscle with relative sparing of the anterior thigh muscles [8].

When a patient’s phenotype is apparently consistent with LGMD-1 or LGMD-4 but unfortunately genetic tests reveal in *CAPN3* missense or splicing variants of uncertain significance, a molecular diagnosis becomes challenging. To assess the effect of novel *CAPN3* variants and thus speculate on their possible pathogenicity, invasive methods requiring muscle biopsy are actually needed. Indeed, Western blotting (WB) and *CAPN3* transcripts analysis from muscle tissue become essential to validate a possible pathogenic variant: CAPN3 reduction in muscle is highly suggestive of pathogenic *CAPN3* variants and only muscle tissue is to date known to express the full-length *CAPN3* isoform.

In order to define possible alternative strategies and to avoid invasive procedures, here we describe novel potential biomarkers for achieving a molecular LGMD diagnosis. With this aim, we describe different *CAPN3* variants, including two novel variants. Through sequencing *CAPN3* transcripts from muscles, fibroblasts and urine, we detected different *CAPN3* isoforms, surprisingly including the full-length transcript. Moreover, we showed for the first time comparable levels of protein and full-length transcript between fibroblasts and muscle tissue, thus supporting the use of skin biopsy as an alternative less invasive method for assessing the amount of CAPN3.

## 2. Materials and Methods

We analyzed the *CAPN3* gene in a cohort of 60 patients with clinical suspicion of LGMD. Selected patients underwent further evaluation including neurological examination, family history collection, electromyography (EMG), muscle biopsy, and skin biopsies for primary fibroblasts isolation. Routine morphology and immunohistochemical study for membranous proteins were performed according to standard procedures. Clinical data including age of onset, distribution of weakness, respiratory condition and cardiac involvement were collected. Morphometric analysis in the available muscle tissues was performed through AxioVision (version 4.5; Zeiss, Oberkochen, Germany). All images were obtained, and the following parameters for each muscle fiber type (I or II) were considered: perimeter, area, and diameter. Statistical analysis was performed by Student’s *t*-test. To unveil differences in the type I/type II ratio, we used a Chi-squared test. The local ethics committee approved this study. All patients provided written informed consent for muscle and skin biopsies and genetic analyses. All the procedures complied with the Helsinki Declaration of 1975.

### 2.1. DNA Analysis

The entire genomic DNA was extracted from specimens deriving from peripheral venous blood sampling using a standard procedure. The *CAPN3* gene (NC_000015.10) was amplified in a pool of 20 different PCR. To perform PCR reactions, we used 50 µL reaction volumes containing 100 ng of template DNA, 0.2 pmol primers, 0.4 mM deoxynucleotide triphosphate, 1.5 mM MgCl2, 1X reaction buffer, and 1.25 U Red Hot DNA Polymerase (Thermo Scientific; Waltham, MA, USA), using a DNA thermal cycler (PTC-200; MJ Research, Waltham, MA, USA). PCR products were sequenced by an automated sequencer ABI 3730 (Applied Biosystems, Foster City, CA, USA). The results were analyzed with Sequence Scanner software (Thermo Fisher Scientific) and compared with the sequence of the human *CAPN3* gene (NG_008660.1). External datasets including 1000 genomes, ExAC and GnomAD, were consulted. A prioritization of the variants was performed as previously described [9]. Putatively deleterious variants were validated by PCR-based standard capillary Sanger sequencing. Novel variants were analyzed by RFLP and in silico analysis through the sequent software tools: Mutation Taster, PolyPhen-2, PROVEAN, snSNPAnalyzer, SIFT, MutPred and PMut.

### 2.2. Fibroblasts Isolation

Skin biopsies obtained from our patients and healthy subjects were washed twice and chopped under sterile conditions for the isolation of primary fibroblasts. After 15 min of trypsin incubation at 37 °C, tissues were placed in 25 mL plastic flasks with 1 mL of Dulbecco’s Modified Eagle’s Medium (DMEM; Gibco, Invitrogen, Carlsbad, CA, USA) supplemented with 10% FCS (Gibco, Invitrogen), 1% L-glutamine (Sigma-Aldrich, Saint Louis, MO, USA) and 1% streptomycin–penicillin (Sigma-Aldrich). After the flasks were incubated overnight at 37 °C with 5% CO_2_, we added 5 mL of supplemented DMEM and cells were grown in standard conditions.

### 2.3. RNA Analysis

The entire RNA isolated from muscle tissue, fibroblasts, buccal swab, and white blood cells (WBCs) of patients and healthy controls was processed using an RNeasy Mini Kit (QIAGEN, Venlo, The Netherlands) according to the manufacturer’s instructions. The RNA from each line underwent DNAse I treatment for 15 min at room temperature (Fermentas, Vilnius, Lithuania). For each sample, 1 µg of total RNA was reverse transcribed using ImProm-II™ Reverse Transcriptase (Promega, Madison, WI, USA) and random oligonucleotides in a 20 µL volume. As previously described [10], in order to analyze the entire coding region of *CAPN3*, the cDNA was amplified into five overlapping regions. We compare the cDNA with the CAPN3 human isoform 1 (NM_000070.2), and we used it for qualitative analysis. The total RNA isolated from urine samples of healthy controls was processed using the ZR Urine RNA Isolation Kit (Zymo Research, Irvine, CA, USA). The concentration and purity of total RNA samples were quantified using the QubitTM RNA IQ assay kit (Thermo Scientific, Waltham, MA, USA).

### 2.4. RT-qPCR

We performed RT-qPCR through QuantiNova SYBR Green RT Mix (Qiagen, Hilden, Germany) according to the manufacturing instructions. The reaction mixture (total volume 20 µL) contained 20 ng of RNA. We conducted all the reactions in triplicates on a CFX96 Real-Time System (Bio-Rad, Hercules, CA, USA). The protocol included 10 min of initial reverse transcription at 50 °C followed by 2 min at 95 °C. Then, we started 40 cycles of 5 s each at 95 °C denaturation and 30 s at 60 °C of primer annealing, extension, and relative fluorescence unit data collection. Data were analyzed with CFX Manager Software V3.0 (Bio-Rad, Hercules, CA, USA). We developed specific isoform assays to detect full-length CAPN3 mRNA in muscle and fibroblast (CAPN3-FW 5′GTGGACAAAGATGAGAAGGCC-3′ and CAPN3-RV 5′TGAGGTTGCAGATCTCCAACT-3′) and validated it to check the reaction efficiency. *CAPN3* expression was compared to the expression of the reference genes. Hypoxanthine phosphoribosyltransferase 1 (HPRT1, qHsaCID0016375) and zinc finger protein 80 (ZNF80, qHsaCED0018708) were used for data normalization. A relative quantification of gene expression was evaluated using the comparative threshold cycle value method 2^−∆∆Ct^.

### 2.5. Western Blotting

The amount of CAPN3 was evaluated by Western blot (WB) analysis of muscle tissue and fibroblasts from both patients and controls. The tissues were lysed on ice with Tissue Protein Extraction Reagent T-PER™ (Thermo Scientific) and Complete Mini Anti-protease Cocktail Tablets (Roche Applied Science, Laval, PQ, Canada) according to the manufacturer’s instructions. Then, 90 μg protein for muscle samples and 40 μg protein for fibroblasts samples were separated by SDS-polyacrylamide gel electrophoresis; afterwards, we transferred electrophoretically the proteins to nitrocellulose membranes (Biorad, Hercules, CA, USA). The membranes were incubated with 1:25 rabbit anti-CAPN3 primary antibody 12A2, (Leica Biosystems, Nussloch, Germany), and 1:15000 peroxidase AffiniPure goat anti-rabbit IgG was used as a secondary antibody (111-035-045, Jackson ImmunoResearch Laboratories, West Grove, PA, USA). GAPDH was used as an internal reference to normalize protein expression. Proteins were detected by ECL SuperSignal^®^ West Pico Chemiluminescent Substrate (Thermo Fisher Scientific) according to the manufacturer’s instructions. The amount of CAPN3 and GAPDH was analyzed by densitometry using ImageJ software (https://rsb.info.nih.gov/ij, accessed on 10 December 2023).

### 2.6. Structural Modeling and Conservativity Analysis

The 3D structure of CAPN3 was generated using human m-calpain (1 kfuL) deposited in the Protein Data Bank and visualized using PyMol (PyMOL v.1.7.0.1; Python v.2.7.2).

Sequence alignments for CAPN3 missense mutations were performed using ClustalW2 to evaluate whether amino acid substitutions occur in conserved regions of the proteins. The following species were used: Homo sapiens, Macaca mulatta, Mus musculus, Rattus norvegicus, Bos taurus, Alligator mississippiensis, Dama dama, Gallus sinae and Xenopus laevis.

## 3. Results

### 3.1. Clinical Features

Among our cohort of 60 LGMD patients, we found potentially interesting *CAPN3* variants in six patients. They harbored seven different *CAPN3* variants, of which two were novel.

Patient 1, 2 and 3 were three siblings born to non-consanguineous parents. Patient 1 was a 59-year-old man showing gait difficulties, easy fatigability and progressive muscle weakness since adolescence. The neurological examination at 59 years of age evidenced waddling gait, calf hypertrophy, scapular winging, severe and symmetrical weakness of shoulder and pelvic girdles muscles (MRC = 2). The EMG examination showed myopathic changes. The level of serum CK was highly increased (2341 U/L). Muscle biopsy showed chronic dystrophic-like features: myofiber diameters variability, atrophic and hypertrophic fibers, fiber splitting, fibrous-adipose replacement, degenerating and/or regenerating fibers, and a predominance of type II fibers. Patient 2, a 56-year-old woman, had been complaining progressive weakness in the biceps brachii muscle of the right arm since she turned 40; then, she experienced diffuse muscle weakness. EMG revealed myopathic changes, and the serum CK level was increased (800-2118 UI/l). The neurological examination was similar to patient 1. Muscle biopsy demonstrated fibers size variability, atrophic fibers, internal nuclei and a predominance of type II fibers. Patient 3, the third sibling, was a 55-year-old woman with progressive muscle weakness starting from 40 years of age, myopathic changes at EMG study and elevated serum CK (1767 UI/l). Neurological examination showed a less severe picture of limb girdle muscle weakness (MRC = 4). Muscle biopsy showed atrophic and hypertrophic fibers without necrosis. The mother of the three siblings was reported to present muscle weakness.

Patient 4 was a 74-year-old woman showing limb girdle weakness from her forties. She showed scapular winging, waddling gait, and severe weakness in the upper and lower girdle muscles. Myopathic changes were detected by the EMG analysis, and serum CK levels were high (1169 UI/l). Muscle biopsy performed at 71 years of age showed increased fiber size variability with 10% central nuclei, diffuse fibrous-adipose replacement and a predominance of type II fibers.

Patient 5 was a 60-year-old man. He referred easy fatigability and progressive limb girdle muscular weakness since adolescence. The neurological examination revealed waddling gait with shoulder weakness (MRC = 3) and pelvic girdles weakness (MRC = 2). Serum CK was 191 Ul/l. Mild variation in fiber size and internal nuclei were evident at muscle biopsy.

Patient 6 was a 51-year-old man referring gait difficulties since 35 years of age and progressive weakness involving limb girdle muscles. He presented showed waddling gait with diffuse muscle wasting and scapular winging. Severe shoulder weakness (MRC = 2) and pelvic girdles involvement (MRC = 2) were also present. Muscle biopsy showed dystrophic changes including increased connective tissue and fiber size variation, internal nuclei, lobulated fibers, predominance of type II fibers, and small-diameter type I fibers. There were also some necrosis and degenerating/regenerating fibers.

### 3.2. Genetic Study and 3D Structure Modeling

**Figure 1:**
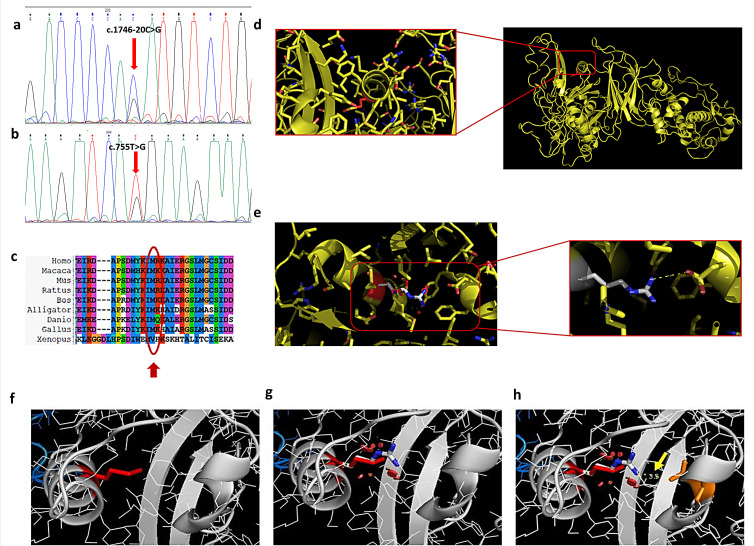
**Met252Arg and c.1746-20C>G variants**. (**a**) Electropherogram of intron 14 highlights the transition C > G in heterozygous state. (**b**) Electropherogram of exon 5 highlights the transition T > G in heterozygous state. (**c**) The alignment of orthologous proteins shows that the variation involves a highly conserved methionine (red arrow). In the 3D structure, the Met252 is shown in red (**d**), which is located at the level of an α-helix of domain II of calpain 3. Substitution of methionine with arginine at position 252 and measurement of the interaction between Arg252 and Asp402 are shown (**e**–**h**). (**f**) Met252, (**g**) Arg252, (**h**) the possible interaction with Arg252 and Asp402 (yellow arrow).

In patients 1 and 2, we found the *CAPN3* variants c.755T > G (p.Met252Arg) and c.1746-20C > G in a compound heterozygous state (Figure 1a,b). Patient 3 showed the heterozygous variant c.755T > G (p.Met252Arg). The variant c.755T > G (p.Met252Arg) was previously described in heterozygous status only in an asymptomatic 48-year-old male with high CK [11]; this variant is absent in the gnomAD and ClinVar database. ACMG criteria suggest a likely pathogenic effect of the variant (PM2, PM5, PP3, PM1, PP2). The alignment of orthologous proteins in the studied species shows that the variation involves a highly conserved methionine (Figure 1c). The p.Met252Arg variant is within an α-helix of the protein, specifically in domain II of calpain 3 (Figure 1d). Similar mutations, situated internally within the three-dimensional structure, can induce alterations in protein folding, leading to variations in molecule stability, particularly when mutations occur within secondary structures rather than loops. Arginine, being an amino acid of considerable size like methionine, does not introduce greater steric hindrance in the 3D structure, as evidenced in Figure 1g. However, unlike methionine, arginine, being a polar, charged amino acid, introduces a positive charge into the structure. This charge is likely neutralized by the nearby negative charge of aspartic acid at position 402. This potential interaction is hypothesized due to the proximity of the two amino acids at a distance of 3.5 Å, which is a distance allowing electrostatic interaction and/or hydrogen bonding (Figure 1h). Such interaction might lead to an increased stabilization of the 3D structure, albeit resulting in one with less negative charge. Therefore, this interaction, by neutralizing the negative charge, may likely perturb the protein function rather than its structure.

**Figure 2:**
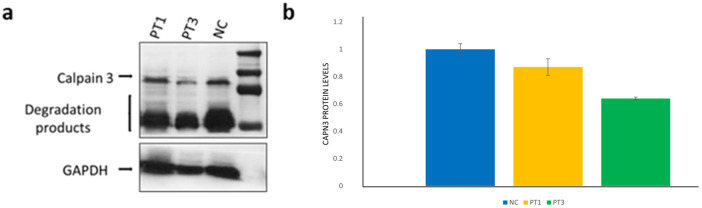
**Western blot analysis in patient 1 and 3.** (**a**) WB showing the expression levels of CAPN3 in muscle tissue of patients 1 (PT1) and 3 (PT3) and one normal control (NC). The bar graph (**b**) shows the expression fold change in CAPN3, calculated by setting the ratio of CAPN3 protein/GAPDH protein band intensities in theNC to 1. The bars show the mean ± SD (data are representative of n = 2 independent experiments).

The c.1746-20C > G variant, laying in intron 14, has been previously reported in several individuals with autosomal recessive LGMD-1 [12,13,14]. However, conflicting evidence has been reported regarding the effect of this variant on splicing [15,16], and ACMG criteria actually suggest a benign effect of the variant (BP7, BP6, BS1, BS2). Our findings through quantitative WB analysis in samples extracted from muscle tissues are reported in Figure 2.

**Figure 3:**
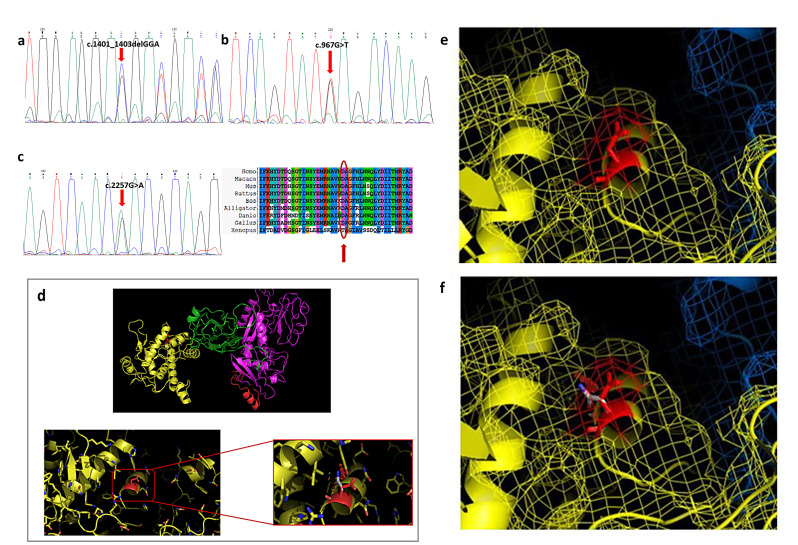
Glu323*, Glu467del, and Asp753Asn variants. (**a**) The electropherogram of exon 11 highlights a deletion of three nucleotides in heterozygous state. (**b**) The electropherogram of exon 7 highlights the G > T transition (red arrow) in heterozygous state. (**c**) The electropherogram of exon 21 highlights the G > A transition (red arrow) in heterozygous state; the alignment of orthologous proteins shows that the variation involves a highly conserved aspartic acid (red arrow). (**d**–**f**) The Asp753Asn variant is shown in red, located at the level of an EF-hand of domain IV of calpain 3. Substitution of aspartic acid with asparagine at position 753 is shown.

Patient 4 showed three previously reported pathogenic mutations: a single base pair mutation encoding a premature stop codon c.967G > T (p.Glu323*) in exon 7 [17,18], a single amino acid deletion c.1401_1403delGGA (p.Glu467del) in exon 11 [11,15], and a missense mutation c.2257G > A (p.Asp753Asn) in exon 21 (Figure 3a–c). Segregation analysis allowed us to establish that mutations c.1395_1397delGGA and c.2257G > A were in the same allele.

The Asp753Asn variant has been reported in the literature in the heterozygous or compound heterozygous state in individuals affected with LGMD without strong evidence for causality and unequivocal conclusions about the association of the variant with the disease [19,20]. The variant is actually classified as uncertain significance in the ClinVar database (accession n° VCV000281081.28).

The Asp753Asn variant leads to the replacement of aspartic acid with asparagine at position 753: this residue is located on the external surface of domain IV of the protein, and this variation probably disrupts the interaction of CAPN3 with possible substrates or with the solvent (Figure 3d–f). As can be seen from the figure, the amino acid variation does not generate steric hindrance, as the two amino acids are very similar to each other; furthermore, the mutation is located on the external surface of the protein and therefore allows greater freedom of positioning of the mutated amino acid. However, the p.Asp753Asn mutation is located in the EF-hand region, essential for the homodimerization of the protein, and variations in this region can disrupt the amino acid interactions for the formation of the dimer. From a chemical point of view, there is the replacement of a charged polar amino acid (aspartic acid) with a non-charged polar one (asparagine): this change in charge could modify the bonds generated with the EF-hand region of another CAPN3 molecule.

Patients 5 and 6 harbored two novel heterozygous CAPN3 variants: c.526G > A (p.Val176Met) and c.2458T > C (p.Tyr820His), respectively.

**Figure 4:**
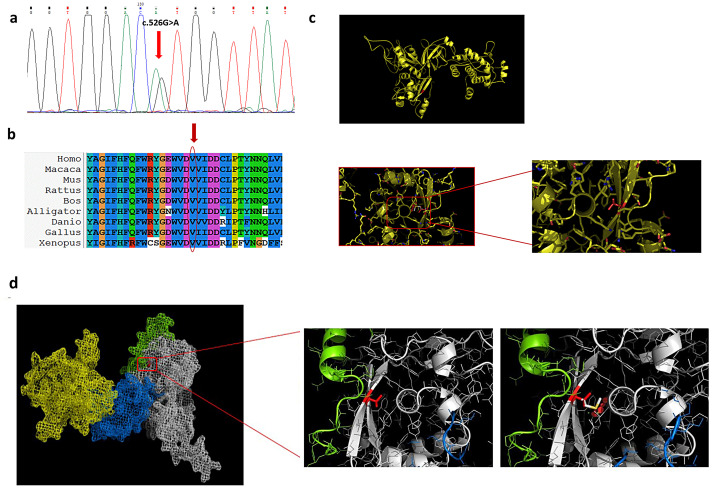
**Val176Met variant.** (**a**) The electropherogram of exon 4 highlights the G > A transition (red arrow) in heterozygous state. (**b**) The alignment of orthologous proteins shows that the variation involves a highly conserved valine (red arrow). PyMOL 3D Structures of the calpain 3. In red, we highlighted the valine at position 176 (**c**,**d**).

According to ACMG criteria, the Val176Met variant is actually classified as a variant of uncertain significance (PP3 PM2 PP2). However, different in silico prediction tools (Mutation Taster, POLYPHEN-2, PROVEAN) suggest its possible damaging effect, and the variant is very rare in the general population (gnomAD, 0.011% allele frequency). The mutagenesis tool in PyMOL shows that the novel variant Val176Met is located within domain II of the protein inside a well-organized region of the 3D structure. This region contains a central α-helix held in position by the surrounding secondary structures. Therefore, the steric hindrance resulting from the amino acid substitution could disrupt the structural organization of this region. Variants like this, within a beta sheet, have a more deleterious impact compared to mutations located in the loops. Both the wild-type and the mutated amino acid, being apolar, do not generate changes in the net charge of the molecule, but they differ in size and therefore steric hindrance: Val176Met generates a strong steric hindrance as the dimensions of methionine are larger than those of valine (Figure 4d). From the structural analysis carried out, the replacement of valine with methionine in position 176 could likely lead to a structural destabilization with a possible consequent functional reduction in the protein.

**Figure 5:**
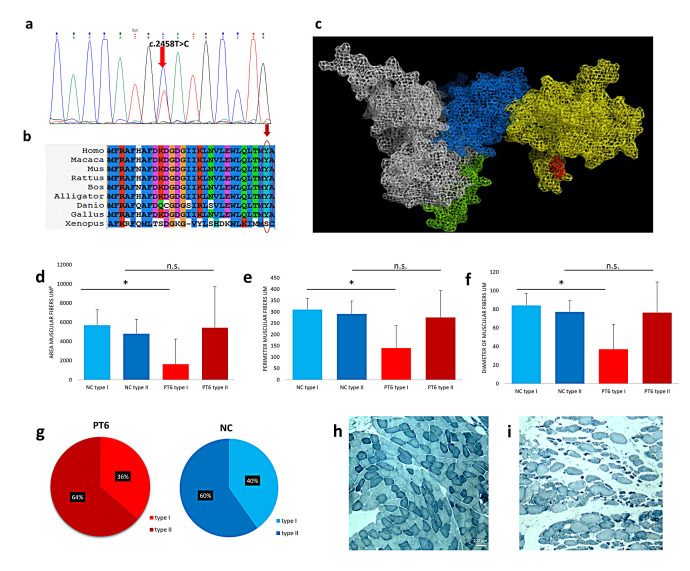
**Tyr820His variant.** (**a**) The electropherogram of exon 24 highlights the T > C transition in heterozygous state. (**b**) Alignment of orthologous proteins shows that the variation involves a highly conserved tyrosine (red arrow). (**c**) Three-dimensional (3D) structure of CAPN3: domain I (green), domain II (white), domain III (blue) and domain IV (yellow). In red, the localization of p.Met819 within EF5-hand of CAPN3 domain IV is shown. Area (**d**), perimeter (**e**) and diameter size (**f**) in type I and type II muscle fibers in a normal control (NC) and PT6. Fibers sizes were measured from muscle sections from a total of 281 fibers in NC muscle and 258 fibers in PT6 muscle. Data represent mean ± SD. Asterisk (*) indicates *p* < 0.05; n.s.: not significant. (**g**) The percentage of type I and type II fibers in an NC and PT6. Muscle biopsy from a normal control (**h**) and PT6(**i**).

The novel c.2458T > C (p.Tyr820His) variant is located in exon 24. ACMG criteria classified this variant as a variant of uncertain significance (PP3, PM2, PP2). However, different in silico prediction tools (Mutation Taster, POLYPHEN-2, PROVEAN) suggest its possible damaging effect, and the variant is absent in gnomAD. Although it was not possible to analyze the Tyr820His variant into the 3D structure since the amino acid substitution is located in a region not perfectly aligned with human m-calpain, the variant is located in the C-terminal region of the protein, which is characterized by high freedom of movement. Therefore, no steric hindrance is generated (Figure 5c), and in the 3D structure, the variant does not generate structural destabilization but probably leads to functional changes. In fact, being located in the EF5-hand, in which histidine introduces a positive charge, it can disturb the homodimerization of calpain 3, since this region is involved in the binding of another calpain 3 molecule. Morphometric analysis performed in patient 6 included 258 mutated muscle fibers and 281 control fibers: there was a significant reduction in diameter, area and perimeter of type I mutated fibers compared to type I control fibers. The percentage of type I fibers in the patient was lower than normal control (36% vs. 40%), although the difference was not statistically significant (Figure 5d–i).

### 3.3. RNA and WB Analysis

**Figure 6:**
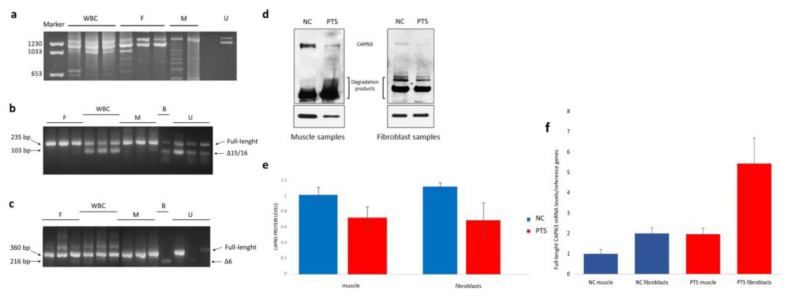
***CAPN3* expression study in multiple tissues through long-range PCR, WB, and RT-qPCR**. Long-range PCR (**a**) to amplify the CAPN3 cDNA region from exons 5 to 17 in white blood cells (WBC), fibroblast (F), muscle (M) and urine (U). PCR amplification of exons 13–17 region (**b**) in fibroblast (F), white blood cells (WBC), muscle (M), epithelial buccal swab (B) and urine (U). PCR amplification of exons 5–8 region (**c**) in fibroblast (F), white blood cells (WBC), muscle (M), epithelial buccal swab (B) and urine (U). Western blot analysis of CAPN3 on muscle and fibroblast samples (**d**) of normal control (NC) and patient 5 (PT5). Histogram bar showing similar protein reduction between muscle and skin (**e**). CAPN3 expression normalized respect to GAPDH expression (data are representative of n = 3 independent experiments). Full-length CAPN3 mRNA expression in muscle tissue and fibroblasts of normal control (NC) and PT5 (**f**). CAPN3 expression in muscle tissue of NC was set to 1. Relative expression levels were calculated relative to HPRT1 and ZNF80 mRNA levels (data are representative of n = 2 independent experiments).

We performed cDNA amplification in healthy controls (Figure 6) and patients using long-PCR encompassing exons 5–17, specifically targeting regions involved in alternative splicing between exons 5–8 and exons 13–17 (Figure 6). Amplification in healthy controls was conducted in samples from muscle tissue, WBCs, fibroblasts, urines, and buccal epithelium. From fibroblast transcripts, we revealed the presence of two variants: the full-length transcript and an isoform deriving from the alternative splicing of exon 6. The urine-derived transcripts showed the presence of the full-length transcript and a transcript lacking exons 15/16. Conversely, buccal epithelium lacked the full-length isoform, displaying only two transcripts: one lacking exon 6 and the other lacking exons 6/15/16. We confirmed in muscle tissue the presence of the full-length *CAPN3* isoform as the unique variant. In WBCs, we confirmed the absence of the full-length isoform and the prevalent presence of four transcripts with an alternative splicing of exons 6/15/16 (Table 1). For each tissue, we were able to identify the different transcripts reported in Table 1 in all the samples tested from controls and patients.

By a quantitative WB analysis of CAPN3 in patient 5, harboring the novel Val176Met variant, we surprisingly found comparable protein levels from fibroblasts and muscle tissue; in particular, the patient showed a 30% protein reduction compared to controls from both tissues (Figure 6d,e).

RT-qPCR showed comparable levels of full-length CAPN3 transcript between muscle tissue and fibroblasts both in controls sample and patient 5 muscle tissue (Figure 6f).

## 4. Discussion

**Figure 7:**
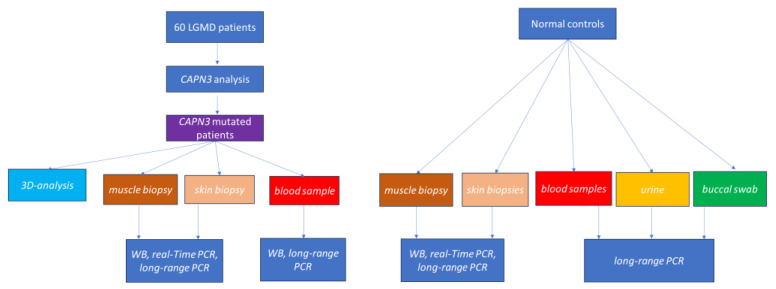
**Flowchart describing the performed analyses.**

Our findings demonstrated for the first time the presence of the full-length *CAPN3* transcript not only in skeletal muscle, as previously known. Through our approach (Figure 7), we surprisingly were able to show the full-length transcript in RNA extracted from human fibroblasts and urines.

Conversely, buccal epithelium lacked the full-length isoform, displaying only two transcripts: one lacking exon 6 and the other lacking exons 6/15/16. Regarding muscle tissue, our findings align with existing literature data, confirming the presence of the full-length isoform as the sole variant. Similarly, we confirmed the expression pattern in WBCs, as previously described by Blasquez et al. [10]. Indeed, during myogenesis, in skeletal muscle, the isoforms without exons 6/15/16 are mainly expressed in myoblasts; in myotubes, the main isoform is the full-length isoform, which is the only isoform recognized in adult tissue to date [21]. In the lymphoblastoid cell line, however, four different isoforms are known to be present: all of them lack exon 15 and may not include exon 6 and/or 16 [10,22].

Our data thus support the less-invasive collection of samples from other tissue than muscle in order to analyze CAPN3 transcript and define the possible pathogenicity of missense or splicing variants.

Moreover, we demonstrated a comparable CAPN3 protein level and full-length transcript levels between muscle and skin samples both in controls and in patients 5. These findings allow us to speculate the use of skin biopsy as an alternative less invasive method to assess the amount of CAPN3 protein when molecular diagnosis turn out to be inconclusive and the hypothesis of calpainopathy is strong.

Among our patients, we detected two novel *CAPN3* variants (c.2458T > C (p.Tyr820His) and c.526G > A (p.Val176Met)). The clinical picture of our patients, and in particular, the histological muscle findings and the 3D prediction study highly support the pathogenic role of these mutations. Moreover, the Val176Met variant determines a marked protein reduction in muscle and fibroblasts, thus supporting the pathogenic role of the variant. The Tyr820His variant represents the most 3′ mutation to date reported in *CAPN3*: it is located in the last EF-hand region of the protein, and it presumably acts as a pathogenic variant, inhibiting the homodimerization of calpain 3.

In CAPN3, four domains (I–IV) and three short inserted sequences (NS, IS1, IS2) are recognized. Although the previously reported mutations span almost the entire length of the gene, a certain clustering around domains II, III, IS1 and IS2 has been shown [23,24]. These mutations primarily affect enzyme activity [23] while other mutations, although preserving the catalytic activity, may affect the ability of CAPN3 to bind to titin [24,25,26]. Some mutations in domain II and most mutations in domain IV hit the ability of CAPN3 to bind to the N2A region and the C terminus of titin, which is an established CAPN3-binding partner [25,27,28]. Mutations in domain III usually alter the Ca^2+^ requirement of CAPN3 [26]. Other studies showed a direct interaction between CAPN3, dysferlin [29], and the dysferlin-interacting protein AHNAK [30]. In particular, Huang et al. [30] demonstrated that AHNAK is cleaved by CAPN3 and showed AHNAK accumulation in the skeletal muscle of CAPN3 mutated patients. Since the protease activity of CAPN3 was associated with domain II, a conserved cysteine protease domain comprising protease core domain 1 (PC1) and PC2 [31], mutation in this domain may be associated with a disturbed interaction with AHNAK and consequently with dysferlin. This may also occur for the mutation located in domain II that we described (p.Met252Arg, p.Glu323*, p.Val176Met).

The c.1746-20C > G variant has been previously reported in several individuals with autosomal recessive LGMD-1 [12,13,14]. However, conflicting evidence has been reported regarding the effect of this variant on splicing, and ACMG criteria actually suggest a benign effect of the variant (BP7, BP6, BS1, BS2). In particular, no splicing abnormalities were previously reported by cDNA analysis of a muscle biopsy from a patient harboring the c.1746-20 C > G and a pathogenic variant, but WB analysis showed reduced protein expression [15]. However, Nascimbeni et al. [16] reported that the variant altered both RNA splicing by cDNA analysis and protein expression by Western blot in a compound heterozygous individual. More recently, Mroczek et al. [32] hypothesized that c.1746-20C > G is a hypomorphic variant with a reduction in RNA and protein expression, and only individuals having a higher ratio of abnormal isoforms are affected. This hypothesis is in line with our results, showing no difference in protein level between patients 1 and 3, respectively, compound heterozygous (Met252Arg and c.1746-20C > G) and heterozygous (Met252Arg); we also showed a milder phenotype in patient 3 compared to the compound’s heterozygous siblings.

A decreased concentration of skeletal muscle calcium release channel RyR1 was previously observed in LGMD-1 muscles, thus suggesting a stabilizing role of calpain for RyR1 [33]. Decreased Ca^2+^ in skeletal muscle can affect CaMKII, which normally activates the transcription factor MEF2 to facilitate the transition from fast (type II) to slow fiber (type I) phenotype. Accordingly, Kramerova et al. [33] showed a reduced percentage of slow fibers in a murine model and abnormalities of type I fibers in *CAPN3* mutated patients. In line with these findings, most of our patients showed a predominance of type II fibers; in addition, the patient harboring the novel p.Tyr820His variant showed a significant reduction in the type I fibers diameter, area, and perimeter. These findings further support the pathogenic role of the described variants.

## 5. Conclusions

In conclusion, our data help to shed light on *CAPN3* expression in multiple tissues, thus supporting the use of novel biomarkers of disease in clinical practice in order to avoid the use of invasive procedures in this rare congenital type of muscle disease.

## Figures and Tables

**Table 1:** Different *CAPN3* transcripts detection in multiple control tissue.

Tissue	Full Length	Δ15	Δ15/16	Δ6/15	Δ6/15/16	Δ6
Muscle (PT1, PT 2, PT3, PT5, PT6, NC = 4)	✔✔					
Fibroblast (PT1, PT2, PT3, PT5, NC = 3)	✔✔					✔✔
Urine (NC = 3)	✔✔		✔✔			
epithelial buccal swab (NC = 2)					✔	✔✔
WBCs (PT1, PT2, PT3, PT4, PT5, PT6, NC = 3)		✔	✔	✔	✔	

Legend: WBCs, white blood cells; NC, normal control, PT, patient; ✔, identified transcript; ✔✔, sequenced band.

## Data Availability

The data that support the findings of this study are available upon reasonable request.
